# Temporal changes in cyclinD-CDK4/CDK6 and cyclinE-CDK2 pathways: implications for the mechanism of deficient decidualization in an immune-based mouse model of unexplained recurrent spontaneous abortion

**DOI:** 10.1186/s10020-022-00523-3

**Published:** 2022-09-01

**Authors:** Zhuo Chang, Hai-xue Kuang, Xueming Zhou, Hui Zhu, Yang Zhang, Yin Fu, Qiang Fu, Bei Jiang, Wei Wang, Sha Jiang, Li Ren, Lei Ma, Xue Pan, Xiao-ling Feng

**Affiliations:** 1 Heilongjiang University of Chinese Medicine, Harbin, Heilongjiang China; 2 First Affiliated Hospital of Heilongjiang University of Chinese Medicine, Harbin, Heilongjiang China; 3 Zhuhai Hospital of Integrated Traditional Chinese and Western Medicine, Zhuhai, China; 4 Hospital of Traditional Chinese Medicine of Qiqihar, Qiqihar, China; 5 Zhaoqing City Guangdong Province Hospital of Traditional Chinese Medicine, Zhaoqing, China; 6 Guang’anmen Hospital, China Academy of Chinese Medicine Sciences, Beijing, China

**Keywords:** Unexplained recurrent spontaneous abortion, Decidualization, Cell cycle

## Abstract

**Background:**

Deficient endometrial decidualization has been associated with URSA. However, the underlying mechanism is poorly understood. This study aimed to investigate the temporal cytokine changes and the involvement of CyclinD-CDK4/6 and CyclinE-CDK2 pathways in the regulation of the G1 phase of the cell cycle during decidualization in a murine model of URSA.

**Methods:**

Serum and decidual tissues of mice were collected from GD4 to GD8. The embryo resorption and abortion rates were observed on GD8 and the decidual tissue status was assessed. In addition, PRL, Cyclin D, CDK6, CDK4, Cyclin E, CDK2 expression in mice were measured.

**Results:**

URSA mice showed high embryo resorption rate and PRL, Cyclin D, Cyclin E CDK2, CDK4, CDK6 down-regulation during decidualization. The hyperactivated Cyclin D-CDK4/CDK6 and cyclin E/CDK2 pathways inhibit the decidualization process and leading to deficient decidualization.

**Conclusion:**

Insufficient decidualization is an important mechanism of URSA. which is related to the decrease of Cyclin D、Cyclin E、 CDK2、CDK4 and CDK6 in decidualization process of URSA.

## Introduction

Recurrent spontaneous abortion (RSA) is defined as two or more consecutive pregnancy losses before 20 weeks of gestation (Zhang et al. 2017). The etiology of RSA is multifactorial, and the influencing factors include anatomical malformations and infections, endocrine dysfunction, prothrombotic, and parental chromosomal disorders (ESHRE Guideline Group 2018; Wilczynski et al. 2012; Kwak-Kim et al. 2010). Unexplained recurrent spontaneous abortion (URSA) accounts for approximately 15% (Santamaria and Taylor 2014) of RSA cases and is characterized as pathological pregnancy with unexplained pathogenesis excluding known factors. Thus, there is an unmet need to explore the mechanism underlying URSA, an unsolved challenge in reproductive medicine.

It has been reported that Decidualization is a crucial link required for the successful establishment and maintenance of pregnancy, moreover, deficient decidualization may be related to URSA (Lee et al. 2013; Fazleabas and Strakova 2002). Decidualization is defined as the cyclic changes the endometrium undergoes in response to stimulation by multiple hormones. During this process, endometrial stromal cells (ESCs) proliferate and transform from spindle-shaped fibroblastic cells into large, round, and multinucleated decidual stromal cells (DSCs) (Lee and DeMayo 2004; Robbert 2020; Okada et al. 2018a). DSCs acquire a secretory epithelioid-like phenotype after transformation and secrete prolactin (PRL), a functional marker reflecting the level of decidualization (Nicole Lustgarten Guahmich 2020; Gellersen et al. 2007). The cellular change as results of decidualization product various growth factors, hormones, cytokines needed to assure an ongoing pregnancy. (Brosens et al. 2009; Klemmt et al. 2006). At present, many studies have proved that pregnancy loss is closely related to deficient decidualization and that this association is ascribed to abnormal changes during decidualization (Laird et al. 2006; Karpovich et al. 2005; Ryan and Taylor 1997; Cha et al. 2012). URSA as a disease characterized by repeated pregnancy loss, may also be caused by insufficient decidualization. Therefore, the exploration of abnormal changes during decidualization in URSA samples may offer new insights into the pathogenesis of this disease and affect the relevant therapeutic approaches.

Decidualization involves a large number of cell cycle events, such as cell proliferation, aging and apoptosis, and this process is believed to be regulated through complex signaling mechanisms orchestrated by cell cycle genes (Okada et al. 2018b; Le et al. 2017). The cell cycle is tightly regulated at two specific checkpoints, namely, the G1-S and G2-M phases. The normal operation of these phases is regulated by a complex interplay of cyclins and cyclin-dependent kinases (CDKs) (Mori et al. 2016). During cell cycle transitions, different cyclins mediate their actions as positive growth regulators by associating with specific CDKs (Das 2010). Cyclin D and cyclin E associate with their specific CDKs, including CDK4, CDK2and CDK6, and this is particularly important for the transition from the G1 to the S phase. The cyclin-CDK complexes orchestrate the cell cycle process by phosphorylating retinoblastoma protein (Rb) as their downstream factor in the cyclin D-CDK4/CDK6 or cyclin E-CDK2 signaling pathway. Some studies have shown that overexpression of cyclin D at the site of implantation improves decidualization defects by shortening the G1 phase and allowing rapid entry into the S phase (Kevin 2007). Furthermore, cyclin E has been proved to play an active regulatory role in decidualization (Roberts 1999a). However, there is still an unmet need to explore the abnormal expression of these Cyclins and CDKs in URSA and the relationship between these changes and the disease.

This study aimed to investigate the temporal changes of cytokines in cyclin D-CDK4/6 and cyclin E-CDK2 pathways during decidualization, and to analyze the effect of these cytokines on this process. Furthermore, we explored the connection between abnormal cytokine changes and the high abortion rate of URSA. The purpose of this study was to further characterize the molecular mechanism underlying URSA.

## Materials and methods

### Animals

30 male DBA/2, 10 male BALB/c and 50 female CBA/J mice (age, 6–8-week-old; weight, 16–22 g) were used in this study. CBA/J mice were purchased from Beijing Huafukang Biotechnology Co., Ltd., and BALB/c and DBA/2 mice were purchased from Beijing Charles River Laboratory Animal Technology Co., Ltd. All animals were housed in our center’s animal facility (Centro de experimental animal, Heilongjiang University of traditional Chinese Medicine) under stable humidity (47%) and temperature (22–24℃) conditions on a 14:10-h light/dark cycle, with free access to drinking water and food. All steps were in full compliance with current regulations on the maintenance and use of experimental animals.

#### Pregnancy model and groups

According to the digital markers in the tail of CBA / J female mouse during adaptive feeding, they were grouped by random number method. 25 virgin CBA /J female mouse were mated with BALB/c male mouse randomly. All the female mouse were examined at 10 am every day after cage closing, focusing on the vaginal plug and sperm in the vaginal smear. CBA / J female mouse with vaginal plug and sperm found in vaginal secretion smear under microscope can be judged as pregnant. The pregnant female mouse after caged with DBA / 2 were regarded as URSA group, while the pregnant female mouse after caged with BALB/c were regarded as NP group. The day the sperm or vaginal plug was observed was considered gestation day 1 (GD1).

#### Sample collection

5 Pregnant female mice were sacrificed on days 4, 5, 6, 7 and 8 of pregnancy. Mice were sacrificed using pentobarbital sodium anesthesia followed by cervical dislocation. At necropsy, uteri were excised, trimmed of fat, washed with saline, and uterine tissues were collected. First, a section of uterus was cut off from the whole uterus and fixed with 4% paraformaldehyde for paraffin section to HE detection. The rest were used to collect decidual tissue. After cutting the bilateral uterine horns longitudinally, expose the endometrial surface and rinse repeatedly until there is no embryo residue under the microscope. After confirming that there was no embryo residue, the decidual tissue samples were obtained and collected. Half of these decidual tissues were stored at -80℃ for RT-PCR, Western-Blot and Elisa test, and the rest were also fixed with 4% paraformaldehyde for preparing the paraffin section of Immunohistochemistry.

### Hematoxylin and eosin (H&E) staining

Decidual tissue samples were taken for detection. The reserved decidual tissues were removed from 4% paraformaldehyde and sectioned, followed by paraffin embedding (2 min). Mouse decidual tissue slides were prepared as 4 μm-thick paraffin sections. The paraffin-embedded sections were deparaffinized with xylene (10 min, twice), and rehydrated using descending ethanol concentrations (100%, 5 min; 95%, 2 min, 80%, 2 min; 70%, 2 min) and running water (2 min). After hematoxylin staining for 10 min, the sections were washed with running water. Differentiation was performed in differentiation medium for 30 s. The stained tissue sections were immersed in warm water (50℃) for 10 min, stained with eosin for 1 min, and washed with running water. After staining with H&E, the sections were dehydrated using an increasing alcohol gradient and cleared with xylene. Finally, the slides mounted with neutral balsam were observed under an Olympus BX60 microscope and photographed with a 3D HISTECH Pannoramic250 Panorama Scanner.

### Elisa

Decidual tissue samples were taken for detection. The reserved decidual tissues were removed from – 80 ℃ refrigerator. After Phosphate Buffered Saline (PBS) was added in proportion to decidual tissue, the tissue was dissociated with a homogenizer and homogenized (500 µl PBS was added for every 10 mg decidual tissue and centrifuged for 20 min at 1509.3 g).), After centrifuged, the supernatant was taken for standby. Standardized operation was carried out according to the operation methods following the instructions provided from the Mouse Prolactin (PRL) ELISA Kit (Cat. E20429, Beijing Cheng Lin, China).

### Immunohistochemistry

Decidual tissue samples were taken for detection. Decidual samples were immediately placed into a 4% paraformaldehyde solution bag and fixed for 10 to 24 h. Paraffin blocks were sectioned into a 5 μm thickness. The EnVision two-step immunohistochemical staining technique was used to detect the expression of PRL in the decidua. The immunohistochemical staining was performed by one person only. Positive controls were included in every batch of tests. PRL (rabbit polyclonal, 1:300, Cat. ab188229, Abcam, UK) was used as the primary antibody. An anti-rabbit HRP/DAP Detection kit (Cat. ab64261, Abcam, UK) was used for primary antibody detection.

### Reverse transcription PCR (RT-PCR)

Decidual tissue samples were taken for detection. The reserved decidual tissues were removed from – 80 ℃ refrigerator. Total RNA was extracted from decidual tissues using the TRIzol Reagent procedure (Cat. 15596026, Thermo Fisher, USA), in accordance with the manufacturer’s instructions. Reverse transcription was performed after the total RNA concentration was determined using 2 μg of total RNA. cDNA was generated using the reverse transcription kit (Cat. RR037A, Takara Bio, China), according to the manufacturer’s instructions. Briefly, RNA was incubated with reverse transcriptase for 15 min at 37 ℃, followed by inactivation of the enzyme at 85 ℃ for 5 s. To detect the target cytokine transcripts, PCR was performed using Takara's 2 × SYBR Green qPCR Mix Kit (Cat. RR820A, Takara Bio, China). The 20 μl reaction system consisted of 1 μl of cDNA, 10 μl of SYBR®Premix ex Taq II (TLI RNaseH plus), 1.6 μl of primers (Table 1) and 7.4 μl of ultrapure water. Cycling conditions consisted of 45 cycles with denaturation steps at 95 ℃ for 30 s, hybridization steps at 95 ℃ for 5 s, and an extension step at 60 ℃ for 30 s. GAPDH was used as endogenous controls for normalization.

**Table 1:** Upstream and downstream primers for target gene detection using PCR

Gene name	Gene primer	Sequence (5ʹ to 3 ʹ)
CDK2	Forward primer	TCCGGATCTTTCGGACTCTG
	Reverse primer	ACAAGCTCCGTCCATCTTCA
CDK6	Forward primer	TTGTGACAGACATCGACGAG
	Reverse primer	GACAGGTGAGAATGCAGGTT
CDK4	Forward primer	CCAGGCAGGCTTTTCATTCA
	Reverse primer	AGGTCCTGGAAGTATGGGTG
Cyclin D	Forward primer	GGGGACAACTCTTAAGTCTCAC
	Reverse primer	CCAATAAAAGACCAATCTCTC
Cyclin E	Forward primer	GAGCTTGAATACCCTAGGACTG
	Reverse primer	CGTCTCTCTGTGGAGCTTATAGAC
GAPDH	Forward primer	GCCTCGTCTCATAGACAAGATGGT
	Reverse primer	GAAGGCAGCCCTGGTAACC

### Western blot analysis

Decidual tissue samples were taken for detection. The reserved decidual tissues were removed from – 80 ℃ refrigerator. Uterine tissues were sufficiently ground with liquid nitrogen and incubated with lysis buffer for 30 min. After centrifugation, the supernatant containing the lysate was collected and stored at 4 °C. After the Protein samples were denatured and protein concentration were determined, the samples separated by SDS-PAGE (Cat. P0014A, Bioworld, China) and transferred to PVDF membranes (Cat. IPVH00010, Millipore, USA). The membranes were, then, blocked with nonfat milk 2 h and incubated with the primary antibodies overnight at 4 ℃. The specific primary antibodies used were as follows: rabbit anti-rat Cyclin E polyclonal antibody (Cat. BS1085, Bioworld, China), rabbit anti-rat Cyclin D polyclonal antibody (Cat. BS2436, Bioworld, China), rabbit anti-rat CDK2 polyclonal antibody (Cat. BS2263, Bioworld, China), rabbit anti-rat CDK4 polyclonal antibody (Cat. MB0027, Bioworld, China), rabbit anti-rat CDK6 polyclonal antibody (Cat. BS6559, Bioworld, China), rabbit anti-rat pRb polyclonal antibody (Cat. bs-1347R, Bioworld, China), rabbit anti-rat GAPDH polyclonal antibody (Cat. ab9485, Abcam, UK) and all were used at a 1:1000 dilution. After washing 3 times, incubated with HRP-conjugated secondary antibodies (goat anti-rabbit IgG (Cat. ZB-2305, ZSGB-BIO, China,1:10,000 dilution) for 1 h at room temperature. The signals were analyzed using an ECL detection Kit (Cat. PE0020, Solarbio, China). After developing and fixing, the film was scanned and analyzed using the Image J 2.0 software to detect the gray values of each protein band.

### Statistical analysis

Statistical analysis was performed using SPSS (version 23.0; IBM Corp.). Normally distributed data are presented as the mean ± SD, and analyzed using independent sample t test. P values of < 0.05 were considered statistically significant. Graphs were generated using PRISM (version 8.0; GraphPad, Inc.).

## Results

### Comparison of the abortion rate and morphology of decidual tissues on GD8

**Fig. 1:**
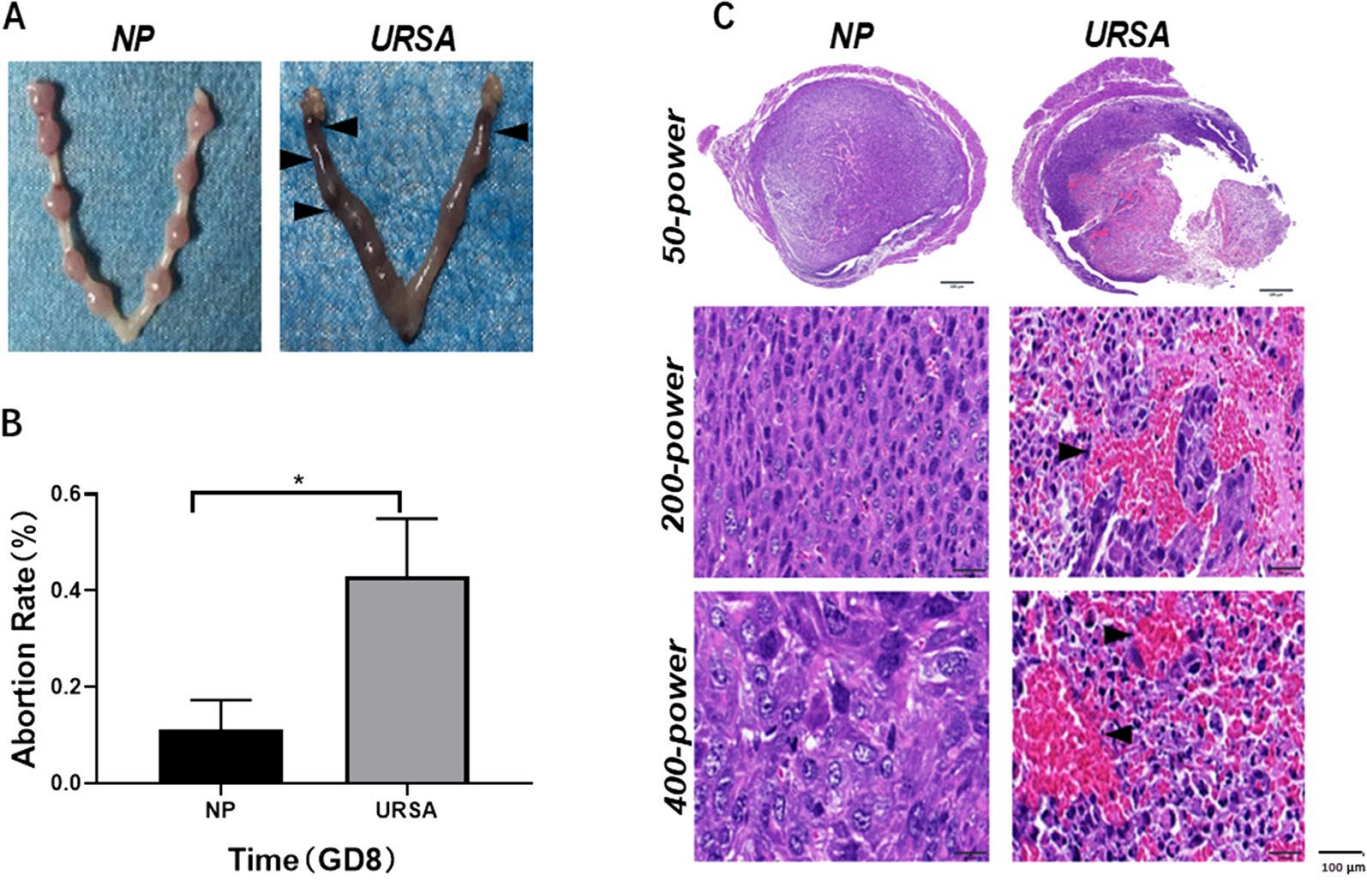
The histomorphology and abortion rate in NP and URSA groups (mean ± SD). **A** On GD8, the uterus of the NP group exhibited normal shape with clearly visible implanted embryos. Immature, absorbed, or dead implanted embryos were often found in the URSA group. Arrowheads point to absorbed and necrotic sites. **B** The abortion rate (mean ± SEM, %) on GD8 was significantly higher in the URSA group compared to that in the NP group. Abortion rate = ratio of absorbed and necrotic embryos to the total number of implanted embryos. *Significantly different (p < 0.005) between null vs. NP. (n = 5 in **A**–**B**); C. 50 × , 200 × , and 400 × magnification images of enucleated decidual tissue stained for hematoxylin and eosin taken after whole tissue scanning. Arrowheads point to pathological form sites. Scale bar, 100 μm. GD8, gestation day 8; NP, normal pregnancy; URSA, unexplained recurrent spontaneous abortion

First, we compared the abortion rates of the NP and URSA groups on GD8. In the NP group, the number of normal embryos was 7–9, almost no absorbed and immature embryos or only one was observed. However, in the URSA group, the total number of embryos was 8–10. However, all embryos were immature, and 3–6 embryos were absorbed or dead (Fig. 1A). The abortion rate on GD8 in the URSA group was (40.48 ± 5.44%), which was significantly higher than that in the NP group (11.07 ± 5.24%) (Fig. 1B). Following H&E staining, the texture of URSA group tissues was loose with uneven staining, and large hemorrhagic and necrotic areas were present. Magnified images of the necrotic area (200 × and 400 ×) showed that the structure of necrotic cells and the boundary between necrotic cells and decidual tissue were unclear (Fig. 1C).

### Temporal changes of PRL quantification in decidual tissues from GD4 to GD8

**Fig. 2:**
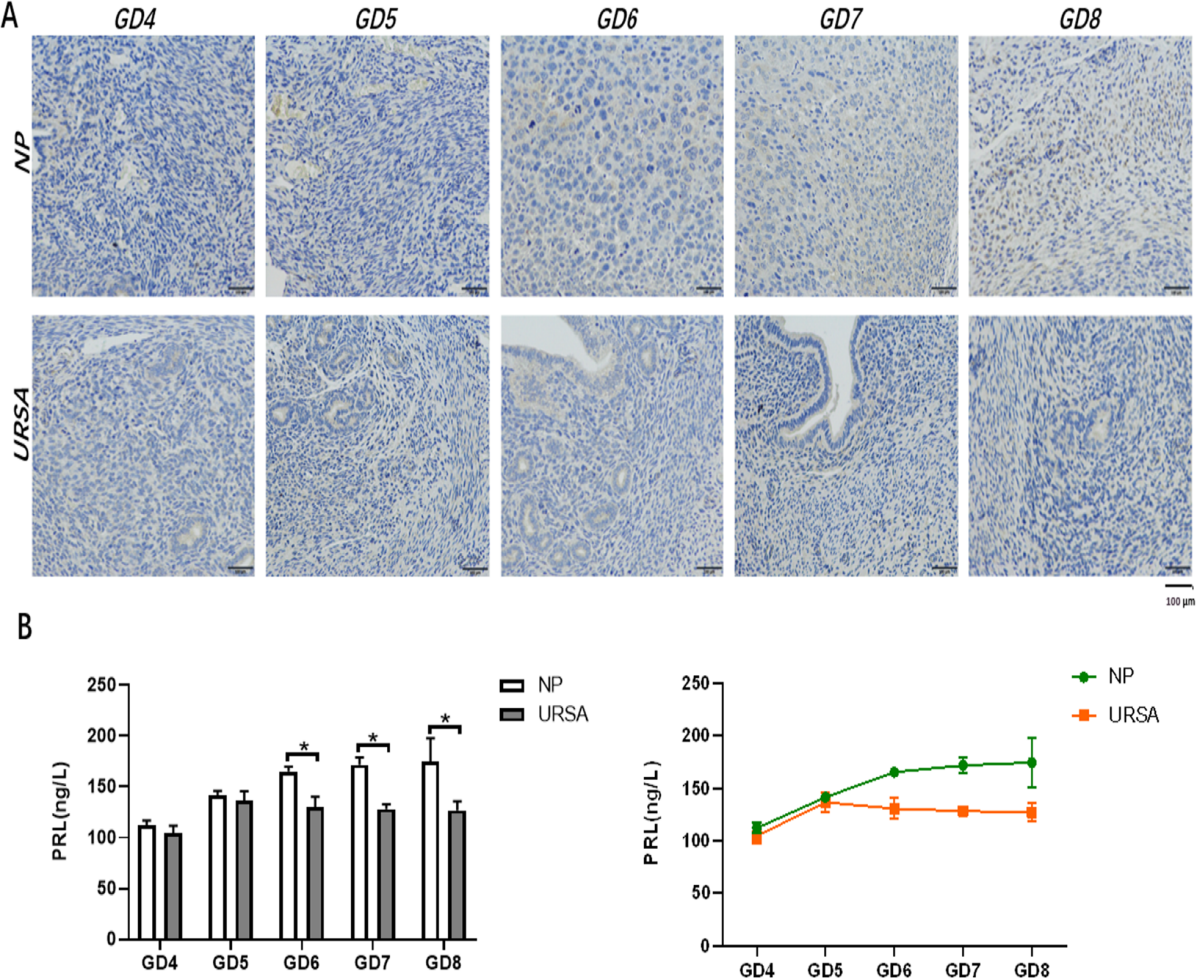
The Temporal changes of PRL quantification in decidual tissues of NP and URSA groups (mean ± SD). **A** Immunostaining for PRL on GD4-GD8. Brown staining indicates positive signals. Nuclei are counterstained with dark brown. The magnifications in the upper panels are at 200 × . Scale bar, 100 μm; **B** The histogram on the left shows the difference in PRL levels by ELISA test in decidual tissues between the two groups on GD4-GD8. (n = 5 in **B**). Results are shown as the mean ± SD. * Significantly different (p < 0.05). The line chart on the right shows the temporal changes of PRL levels. Unit (ng/L) means protein concentration

In order to better observe the temporal changes of PRL protein expression during decidualization in Ursa mice, we detected the temporal and spatial expression of PRL protein from gd4 (decidualization start time) to gd8 (decidualization end time) in two groups of tissues. As shown in Fig. 2A, brown staining is PRL positiveand. Compared with the expression of PRL in decidua of the same gestational days, the PRL expression in NP group was higher than that in URSA group. We found the expression of PRL showed a increased tendency according to pregnancy days in NP group, especially in GD6-GD8. However the PRL expression did not change significantly in URSA group. These results can be observed directly according to the immunohistochemical results. In order to compare the PRL quantification in two groups accurately, we used ELISA method.

The results of ELISA showed that the quantification of PRL in the NP and URSA groups was similar on GD4 and GD5, an early time of decidualization. However, unlike the continuous increase of PRL quantification in the NP group, PRL quantification in the URSA group decreased slightly after GD6 and until GD8, the middle and late stages of decidualization. Furthermore, the PRL levels of the two groups were highest on GD6 and a significant difference was apparent from GD6 to GD8. Finally, the URSA decidual tissue exhibited decidualization deficiency.

### Cyclin-CDK expression during decidualization

**Fig. 3:**
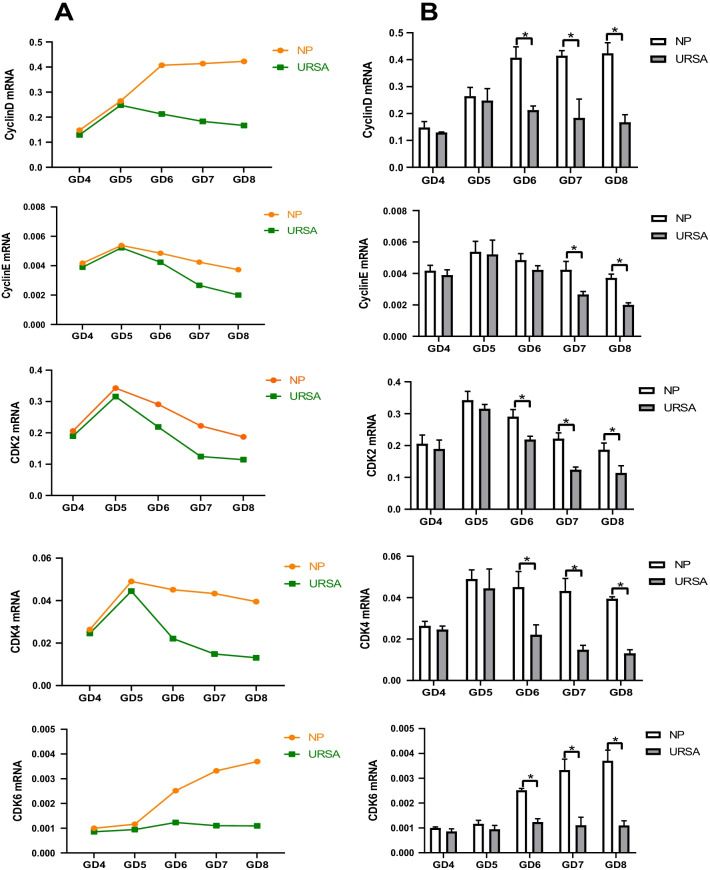
The indexes gene expression of Cyclin-CDK in decidual tissues of NP group and URSA groups (mean ± SD). Quantitative RT-PCR analyses of the cell cycle regulatory genes cyclin D, cyclin E, CDK2, CDK4, CDK6 and of the control gene GAPDH from GD4-GD8 in the NP and URSA groups. **A** The line charts on the left show the temporal changes of gene expression from GD4 to GD8. **B** The bar graphs on the right show the mRNA expression difference in decidual tissues between the two groups on GD4-GD8. (n = 5 in **A**–**B**). Results are shown as the mean ± SD. * Significantly different (p < 0.05)

**Fig. 4:**
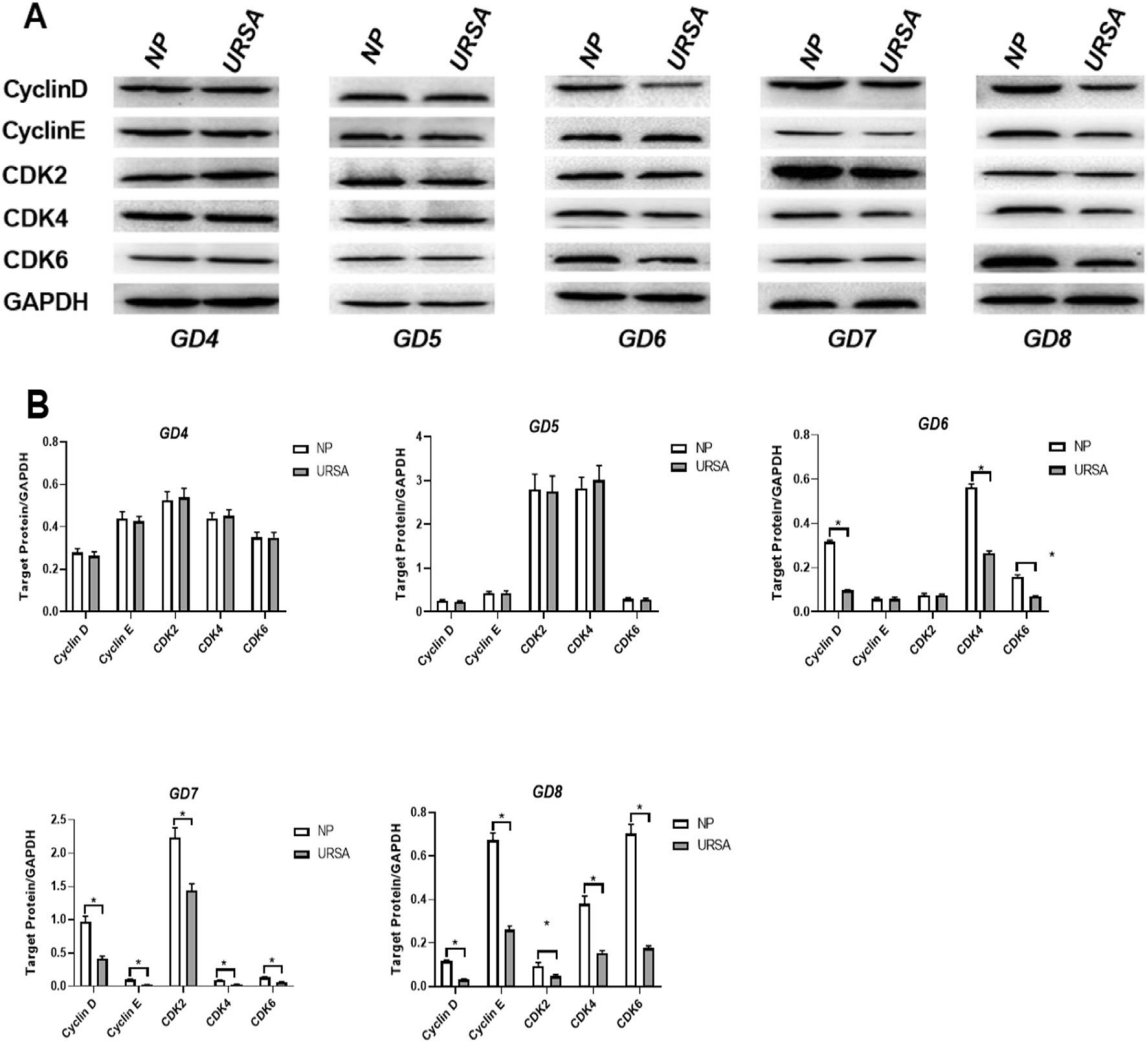
The indexes gene expression of Cyclin-CDK in decidual tissues of NP group and URSA groups (mean ± SD). **A** Western blot analyses of cyclin D, cyclin E, CDK2, CDK4, CDK6 and GAPDH in decidual tissues from GD4 to GD8 between the NP and URSA groups. **B** Bar graphs showing the quantitative analysis of the protein expression levels of these cytokines based on the Western blot analyses. (n = 5 in **A**–**B**). Results are shown as the mean ± SD. *Significantly different (p < 0.05)

To determine the cause of deficient decidualization in URSA mice, we measured the levels of cell cycle regulatory genes in decidual tissues throughout the whole decidualization process. The results revealed that these genes played an important role in decidualization. Firstly, we found that the gene levels of the NP and URSA groups showed different trends mainly after GD5. The cyclin D levels of the NP group tended to be stable after reaching their peak on GD6, whereas those in the URSA group gradually decreased after GD5. The CDK6 levels of the NP group gradually increased from GD4 to GD8, whereas those of the URSA group were relatively stable and did not significantly increase. Although the levels of cyclin E, CDK4 and CDK2 mRNA in the two groups gradually decreased after reaching their peak on GD5, the degree of decline in the URSA group was significantly greater than that in the NP group (Fig. 3A). Further, by comparing the time of significant differences in these indicators, we found that except for the significant difference between the two groups in the cyclin E mRNA levels beginning on GD7, significant differences in the mRNA expression of other genes began on GD6 (Fig. 3B). In general, the gene level differences between the URSA and NP groups occurred in the middle and late stages of decidualization. Consistent with the above results, further quantitative analyses of cell cycle regulatory genes (cyclin D, cyclin E, CDK2, CDK4, CDK6) using western blotting also revealed significant differences between the two groups in the levels of all target proteins on GD7-GD8, whereas only cyclin D, CDK4 and CDK6 levels showed significant differences on GD6 (Fig. 4).

## Discussion

URSA is a common disease that seriously threatens the reproductive safety of women. Its pathogenesis is complex and is an urgent problem in the field of reproduction worldwide. Therefore, exploring the pathogenesis of URSA from multiple perspectives and new directions is conducive to promoting the development of global life and health. Among the many mechanisms that may cause URSA disease, decidualization deficiency has aroused interest. A wealth of evidence has confirmed that insufficient decidualization is the key cause of a series of adverse pregnancy outcomes, and the internal mechanism of abortion may be related to the morphological or functional defects of the decidua, which is caused by insufficient decidualization (Ng et al. 2020; Blois et al. 2010; Achache et al. 2010). However, decidualization is a dynamic process with time continuity; therefore, the lack of decidualization is only a result. This points to the problems in the decidualization process as the key concern. The decidualization process of mice lasted for 8 days, and the decidualization reaction was initiated after blastocyst implantation on GD4. GD4 to GD6 is the early and middle stage of decidualization. During this period, ESCs around the blastocyst differentiated into DSCs and proliferated continuously until they formed the primary decidual zone (PDZ) surrounding the blastocyst. Subsequently, from GD7 to GD8 (the late stage of decidualization), the cells in the PDZ continued to proliferate and finally formed a secondary decidual zone (SDZ) wrapped in the outer layer of the PDZ (Okada et al. 2018b; Lustgarten Guahmich et al. 2020; Wang and Dey 2006; Cha et al. 2014; Das 2009). Thus far, the 4-day decidualization process was successful, and the mouse endometrium was completely transformed into pregnant decidua. Previous studies have shown that many cell proliferation events are involved in the decidualization process, and cell proliferation needs to be regulated by Cyclins and CDKs. The cell proliferation cycle needs to go through the G1 (first gap), S (synthesis), G2 (second gap) and M (mitosis) phases. In the four phases, the cell stays in the G1 phase the longest; this plays a key role in the whole process of cell proliferation (Roberts 1999b; Schafer 1998; Poon 2016). In addition, cells are easily blocked in the G1 phase, and this affects the cell proliferation process. Fortunately, the cell cycle block in the G1 phase is recoverable and can re-enter the cell cycle process after being re-stimulated. The key cyclins and CDKs that determine whether cells stagnate in G1 phase or quickly enter the next phase are Cyclin D, Cyclin E, CDK4, CDK6 and CDK2 (Sherr and Roberts 1999; Thoma et al. 2021). Cyclin D accumulates massively in the G1 phase and forms cyclin D/CDK complexes after binding with CDK4 or CDK6, which can accelerate the initiation of DNA replication, while Cyclin E binding with CDK2 can also promote DNA replication. These Cyclin /CDK complexes play an important role in promoting cells from phase G1 to S. However, when the decreased content of the above indicators leads to decreased Cyclin /CDK complexes, it causes the cell to stagnate in the G1 phase and hinder the process of cell proliferation (Wang et al. 2018; Liu et al. 2017). Ultimately, this study raised the following questions: what abnormalities occurred during decidualization, resulting in insufficient decidualization? Is this condition related to the abnormal process of decidualization?

To provide answers, we conducted small size animal sample to preliminary experimental research. This study selected the internationally recognized URSA animal model (CBA/J female mice were caged with DBA/2 male mice, and CBA/J female mice were considered URSA animal models after pregnancy). CBA / J mice first appeared in 1920. It came from the mating of Bagg female mice and DBA male mice, so it may be easier to mate with DBA mice, and there will be repeated abortion due to inbred lines after mating (Bonney and Brown 2014). Firstly, we detected PRL (a decidualization marker, which can be used as an index to reflect the degree of decidualization) content in decidua tissues of the two mice groups on GD8 to compare the correlation between the final decidualization level and URSA. The results showed that the content of PRL in the decidua of URSA mice was significantly lower than that of normal mice, which confirmed a correlation between incomplete decidua and URSA occurrence. This finding was also consistent with previous studies. Next, to further explore this phenomenon, we detected some indexes in the whole process of decidualization (GD4-GD8) of the two mice groups. Our detection results showed that during decidualization of normal female mice, the change trend of Cyclin E and CDK2 was similar. Their expression in decidua began to rise on GD4 and declined after reaching the peak on GD5 to GD8. This result is also consistent with previous research results (Das 2009; Tan et al. 2002). The expression of Cyclin D continued to rise during decidualization, increased significantly from GD4 to GD6, and became flat from GD7-GD8. The level of CDK4 increased briefly on days 4 and 5 of pregnancy and then decreased gradually. However, CDK6 showed the opposite trend to CDK4; the expression of CDK6 was low on GD4 and GD5 and increased from GD6-GD8. Next, we compared the differences between URSA mice and normal mice. The results showed that compared with normal mice, the content of the above indexes in URSA mice decreased in varying degrees during decidualization. Although the trend of Cyclin D, CDK4, and CDK6 in URSA mice was consistent with that of normal mice, their expression levels were lower, and the difference was more obvious after the 5th day of pregnancy. In addition, compared with normal mice, the expression of Cyclin E and CDK2 in URSA mice showed a more significant downward trend after GD5. According to the test results of this study and previous findings (Yu et al. 2020; Logan et al. 2012), our analysis showed that normal mice had accumulated a certain amount of Cyclin E and CDK2 in the endometrium before implantation on GD4, which can accelerate the process of cell cycle, promote the proliferation of ESC, and increase endometrial receptivity under the synergistic action of other factors; this is conducive to embryo implantation. After the decidualization reaction induced by embryo implantation, the levels of Cyclin E and CDK2 increased on GD5. Cyclin D and CDK4 also showed an upward trend from day 4−5 of pregnancy. Cyclin and CDK complexes worked together to promote the cell cycle process around the embryo implantation site to accelerate the cell proliferation, quickly form PDZ, and wrap the blastocyst. Furthermore, this study found that the decidualization marker PRL also rose rapidly. After GD6, with PDZ and SDZ formation on GD7 and GD8, Cyclin D in PDZ almost disappeared and inhibited Cyclin/CDK complex activity; thereby affecting the cells from phase G1 to S. However, because the level of Cyclin D in SDZ remained high, that of Cyclin D in decidua rose slightly, while the gradual rise of CDK6 compensated for the CDK4 decline in order to maintain the activity of Cyclin D/CDK complexes. Although some decidual cells can continue to proliferate, some cells return to the G1−S phase. These cells break away from the cell proliferation cycle and continue to replicate in the nucleus, which can maintain the stability of genetic genes, limit the life span of decidual cells, and mediate their orderly apoptosis, thus providing space for embryonic growth. Therefore, this study found that Cyclin/CDK complex in the decidualization process of normal pregnant female mice showed an overall upward trend, conducive to maintaining pregnancy. This result also reveals that the periodic changes of cells occur continuously in the process of decidualization, and the cytokines regulating G1 phase also change dynamically with time. Therefore, to answer the question: what changes have taken place in the decidualization process of URSA, which eventually leads to the lack of decidualization? Our findings speculate that although the Cyclin E and CDK2 in the decidua of URSA model mice are slightly lower than those of normal mice during the peri-implantation period and early pregnancy of 4, 5 or even 6 days (the formation time of PDZ), this cannot severely hinder the process of decidualization. Moreover, the level of decidualization marker PRL did not decrease significantly. However, with the advancement of the decidualization process to GD7 and GD8 of pregnancy (formation time of SDZ), the decidualization level of URSA mice decreased significantly compared with normal mice after the continuous decline of Cyclin E and CDK2 levels. In addition, the levels of Cyclin D and CDK4 also showed a downward trend after the formation of PDZ on GD6-GD8, and CDK6 did not increase during the same time like normal female mice but showed a continuous downward trend during the whole decidualization process. This could not compensate for the maintenance of Cyclin D/CDK complexes activity and would affect the cells from entering the next cycle stage, hinder the decidualization process, and affect the formation of PDZ and SDZ, which will cause decidualization defect, affect the function of pregnant decidua, and eventually lead to abortion. Although our experiment used a small size sample of animal research, it is of great significance to explore the pathogenesis of URSA and guide clinical treatment. Based on the regulation of Cyclin / CDK network to improve the degree of decidualization, it may become a potential therapeutic target for the treatment of URSA, and also can enrich the therapeutic means of URSA.

In conclusion, through the observation of the whole process of decidualization in animal models, this study found that incomplete decidualization is an important cause of URSA, and the abnormal changes of Cyclin D, Cyclin E, and protein-dependent kinases CDK2, CDK4 and Cdk6 during decidualization, especially their abnormal decrease in the middle and late stages of decidualization, are closely related to the incomplete decidualization of URSA mice. However, the mechanism remains unclear completely. Therefore, further in-depth exploration and research at the cellular level are required.

## Data Availability

The datasets used and/or analyzed during the current study are available from the corresponding author on reasonable request.

## Abbreviations

RSA: Recurrent spontaneous abortion
URSA: Unexplained recurrent spontaneous abortion
CDK: Cyclin D-cyclin-dependent kinase
NP: Normal pregnant
GD4: Gestation day 4
GD5: Gestation day 5
GD6: Gestation day 6
GD7: Gestation day 7
GD8: Gestation day 8
ESCs: Endometrial stromal cells
DSCs: Decidual stromal cells
PRL: Prolactin
H&E: Hematoxylin and eosin
RT-PCR: Real time-PCR
PVDF: Polyvinylidene fluoride
TBST: Tween-20
GAPDH: Glyceraldehyde-3-phosphate dehydrogenase
PDZ: Primary decidual zone
SDZ: Secondary decidual zone
G Phase: Interphase
M Phase: Division phase
G1 Phase: First gap
S Phase: Synthesis
G2 Phase: Second gap

## References

[CR1] Achache H, Tsafrir A, Prus D (2010). Defective endometrial prostaglandin synthesis identified in patients with repeated implantation failure undergoing in vitro fertilization. Fertil Steril.

[CR2] Berkhout RP, Lambalk CB, Repping S, Hamer G, Mastenbroek S (2020). Premature expression of the decidualization marker prolactin is associated with repeated implantation failure. Gynecol Endocrinol..

[CR3] Blois SM, Klapp BF, Barrientos G (2010). Decidualization and angiogenesis in early pregnancy: unravelling the functions of DC and NK cells. J Reprod Immunol.

[CR4] Bonney EA, Brown SA (2014). To drive or be driven: the path of a mouse model of recurrent pregnancy loss. HHS Author Manuscripts.

[CR5] Brosens JJ, Parker MG, McIndoe A, Pijnenborg R, Brosens IA (2009). A role for menstruation in preconditioning the uterus for successful pregnancy. Am J Obstet Gynecol.

[CR6] Cha J, Sun X, Dey SK (2012). Mechanisms of implantation: strategies for successful pregnancy. Nat Med.

[CR7] Cha J, Bartos A, Park C (2014). Appropriate crypt formation in the uterus for embryo homing and implantation requires Wnt5a-ROR signaling. Cell Rep.

[CR8] Das SK (2009). Cell cycle regulatory control for uterine stromal cell decidualization in implantation. Reproduction.

[CR9] Das SK (2010). Regional development of uterine decidualization: molecular signaling by Hoxa-10. Mol Reprod Dev.

[CR10] ESHRE Guideline Group on RPL, Bender Atik R, Christiansen OB, Elson J, Kolte AM, Lewis S, Middeldorp S, Nelen W, Peramo B, Quenby S, Vermeulen N, Goddijn M, ESHRE guideline: recurrent pregnancy loss. Hum Reprod Open. 2018;2018(2):hoy004. doi:10.1093/hropen/hoy004. 10.1093/hropen/hoy004 PMC6276652 31486805

[CR11] Fazleabas AT, Strakova Z (2002). Endometrial function: cell specific changes in the uterine environment. Mol Cell Endocrinol.

[CR12] Gellersen B, Brosens IA, Brosens JJ (2007). Decidualization of the human endometrium: mechanisms, functions, and clinical perspectives. Semin Reprod Med.

[CR13] Guahmich NL, Farber G, Shafiei S, McNally D, Redmond D, Kallinos E, Stuhlmann H, Dufort D, James D, Blobel CP (2020). Endothelial deletion of ADAM10, a key regulator of Notch signaling, causes impaired decidualization and reduced fertility in female mice. Angiogenesis.

[CR14] Karpovich N, Klemmt P, Hwang JH, McVeigh JE, Heath JK, Barlow DH, Mardon HJ (2005). The production of interleukin-11 and decidualization are compromised in endometrial stromal cells derived from patients with infertility. J Clin Endocrinol Metab..

[CR15] Klemmt PA, Carver JG, Kennedy SH, Koninckx PR, Mardon HJ (2006). Stromal cells from endometriotic lesions and endometrium from women with endometriosis have reduced decidualization capacity. Fertil Steril.

[CR16] Kwak-Kim J, Park JC, Ahn HK, Kim JW, Gilman-Sachs A (2010). Immunological modes of pregnancy loss. Am J Reprod Immunol.

[CR17] Laird SM, Tuckerman EM, Li TC (2006). Cytokine expression in the endometrium of women with implantation failure and recurrent miscarriage. Reprod Biomed Online.

[CR18] Le A, Wang Z, Dai X, Xiao T, Zhuo R, Zhang B, Xiao Z, Fan X (2017). Icaritin inhibits decidualization of endometrial stromal cells. Exp Ther Med.

[CR19] Lee KY, DeMayo FJ (2004). Animal models of implantation. Reproduction.

[CR20] Lee KY, Jeong J-W, Wang J, Ma L, Martin JF, Tsai SY, Lydon JP, DeMayo FJ (2007). Bmp2 is critical for the murine uterine decidual response. Mol Cell Biol.

[CR21] Lee CH, Kim TH, Lee JH, Oh SJ, Yoo JY, Kwon HS, Kim YI, Ferguson SD, Ahn JY, Ku BJ, Fazleabas AT, Lim JM, Jeong JW (2013). Extracellular signal-regulated kinase 1/2 signaling pathway is required for endometrial decidualization in mice and human. PLoS ONE.

[CR22] Liu SL, Liu Z, Zhang LD (2017). GSK3β-dependent cyclin D1 and cyclin E1 degradation is indispensable for NVP-BEZ235 induced G0/G1 arrest in neuroblastoma cells. Cell Cycle.

[CR23] Logan PC, Steiner M, Ponnampalam AP, Mitchell MD (2012). Cell cycle regulation of human endometrial stromal cells during decidualization. Reprod Sci.

[CR24] Lustgarten Guahmich N, Farber G, Shafiei S (2020). Endothelial deletion of ADAM10, a key regulator of Notch signaling, causes impaired decidualization and reduced fertility in female mice. Angiogenesis.

[CR25] Mori M, Bogdan A, Balassa T, Csabai T, Szekeres-Bartho J (2016). The decidua—the maternal bed embracing the embryo—maintains the pregnancy. Semin Immunopathol.

[CR26] Ng S-W (2020). Endometrial decidualization: the primary driver of pregnancy health. Int J Mol Sci.

[CR27] Okada H, Tsuzuki T, Murata H (2018). Decidualization of the human endometrium. Reprod Med Biol.

[CR28] Okada H, Tsuzuki T, Murata H (2018). Decidualization of the human endometrium. Reprod Med Biol.

[CR29] Poon RY (2016). Cell cycle control: a system of interlinking oscillators. Methods Mol Biol.

[CR30] Roberts JM (1999). Evolving ideas about cyclins. Cell.

[CR31] Roberts JM (1999). Evolving ideas about cyclins. Cell.

[CR32] Ryan IP, Taylor RN (1997). Endometriosis and infertility: new concepts. Obstet Gynecol Surv.

[CR33] Santamaria X, Taylor H (2014). MicroRNA and gynecological reproductive diseases. Fertil Steril.

[CR34] Schafer KA (1998). The cell cycle: a review. Vet Pathol.

[CR35] Sherr CJ, Roberts JM (1999). CDK inhibitors: positive and negative regulators of G1-phase progression. Genes Dev.

[CR36] Tan J, Raja S, Davis MK, Tawfik O, Dey SK, Das SK (2002). Evidence for coordinated interaction of cyclin D3 with p21 and cdk6 in directing the development of uterine stromal cell decidualization and polyploidy during implantation. Mech Dev.

[CR37] Thoma OM, Neurath MF, Waldner MJ (2021). Cyclin-dependent kinase inhibitors and their therapeutic potential in colorectal cancer treatment. Front Pharmacol.

[CR38] Wang H, Dey SK (2006). Roadmap to embryo implantation: clues from mouse models. Nat Rev Genet.

[CR39] Wang Z, Wang Y, Wang S (2018). Coxsackievirus A6 induces cell cycle arrest in G0/G1 phase for viral production. Front Cell Infect Microbiol.

[CR40] Wilczynski JR, Radwan P, Tchorzewski H, Banasik M (2012). Immunotherapy of patients with recurrent spontaneous miscarriage and idiopathic infertility: does the immunization-dependent Th2 cytokine overbalance really matter?. Arch Immunol Ther Exp.

[CR41] Yu D, Liu Q, Qiao B, Jiang W, Zhang L, Shen X, Xie L, Liu H, Zhang D, Yang B, Kuang H (2020). Exposure to acrylamide inhibits uterine decidualization via suppression of cyclin D3/p21 and apoptosis in mice. J Hazard Mater.

[CR42] Zhang M, Jiawei X, Bao X, Niu W, Wang L, Linqing D, Zhang N, Sun Y (2017). Association between genetic polymorphisms in interleukin genes and recurrent pregnancy loss—a systematic review and meta-analysis. PLoS ONE.

